# The Effect of Work Connectivity Behavior After-Hours on Employee Psychological Distress: The Role of Leader Workaholism and Work-to-Family Conflict

**DOI:** 10.3389/fpubh.2022.722679

**Published:** 2022-02-23

**Authors:** Mingchao Dong, Tianlu Zhang, Yingwu Li, Zhengzheng Ren

**Affiliations:** ^1^Department of Psychology, Renmin University of China, Beijing, China; ^2^The Laboratory of Department of Psychology, Renmin University of China, Beijing, China

**Keywords:** work connectivity behavior after-hours, work-to-family conflict, leader workaholism, psychological distress, mental health

## Abstract

**Background:**

The work connectivity behavior after-hours (WCBA) has become increasingly intense among Chinese employees in recent years, especially in the rapidly developed internet industry. This has made the after-hours work connectivity behavior, a popular topic in the organizational psychology field. Based on boundary theory, we explored the mechanism of after-hour work connectivity behavior on employees' psychological distress and identified the work-to-family conflict (WFC) as mediator. Besides, leader characteristics are essential environmental variables and always play as moderators, among which leader workaholism is prevalent in the internet industry. However, the impact of leader workaholism on employees' behavior is still inconsistent and even contradictory. Thus, this study further examines the moderating effect of leader workaholism between the after-hour work connectivity behavior and employees' psychological distress.

**Methods:**

We conducted a multitime, multisource questionnaire survey in Internet companies in China. Before collecting the data, all participants were assured that their responses would be confidential and used only for academic research. At time 1, the team leader rated his or her workaholism, and team members rated WCBA. At time 2 (3 weeks later), team members were asked to complete the questionnaire containing scales of WFC, psychological distress. The two rounds of data collection resulted in 211 matched team leader–team member responses. We performed a path analysis using Mplus 7.4.

**Results:**

Both the duration and frequency of WCBA can positively predict employees' psychological distress through WFC (the mediating effect = 0.628, 95% CI = [0.593, 0.663]). Specifically, WCBA can increase the level of WFC, which leads to the employees' psychological distress further. Leader workaholism can negatively moderate the relationship between WCBA and WFC, further moderating the mediating effect of WFC.

**Conclusions:**

Work-to-family conflict played as a mediator in the relationship between WCBA and employees' psychological distress. These results may be helpful to recognize the negative effect of WCBA and the role of leader workaholism in the relationship.

## Introduction

The development of information technology has intensified the competition in the capital market and made the Internet field a high-pressure industry, which has created more overtime work and more flexible working systems. Some organizations hope that their employees can keep online for their job during off-work hours so that they could be prepared to work at any time. This typical work connectivity behavior after-hours (WCBA) often leads to individuals' physical fatigue and psychological distress. It is also an invasion of family time, which leads to the breakdown of work–family balance. Drawing on the work–family boundary theory, work-to-family conflict (WFC) can cause employees' psychological distress. However, there is seldom literature exploring the relationship between WCBA and employees' psychological distress from the work–family boundary perspective. This study aims to address this gap by introducing WFC as a mediator based on work–family boundary theory. Besides, leader characteristics are important environmental variables. In the Chinese internet industry, leader workaholism is prevalent. How this environmental variable works on WFC and employees' psychological distress are also one question of this study.

## Theoretical Background and Hypothesis

### WCBA and Psychological Distress of Employees

In this study, WCBA refers to employees participating in work in any place outside of working hours. According to the boundary theory, the impact of work connectivity behavior on individual health during non-working hours can be divided into work and family interfaces. On the work interface, employees will suffer from physical and mental exhaustion, emotional exhaustion, job burnout (1), a decrease in job satisfaction when they experience longtime non-working hours' connections. They would even have the psychological tendency to try to disengage from work (2). Ragsdale and Hoover (3) investigated the relationship between WCBA and employees' emotional exhaustion and found that using Internet communication tools (ICTs) to deal with work during non-working hours increases individual emotional exhaustion and role stress. At the family interface, according to the allostatic load theoretical model (4), dealing with excessive work pressure for a long time will have dual impacts on employees' mental and physical health and eventually increase the occurrence of depression and cardiovascular diseases. Li et al. (5) used the job demand and control (JDC) model to study the employees of a company in state grid. They found that the increase of working hours per week had a positive prediction on occupational stress and depressive symptoms measured by the PHQ-9 scale. When employees worked more than 60 h per week, their health risks increased significantly, and excessive or frequent connectivity during nonworking time consumes a large number of mental resources and affects self-recovery (6). Overall, being occupied by work in the leisure time would hurt individuals' physical and mental health, which is also known as psychological distress. Thus, we propose the following hypothesis:

*Hypothesis 1*: WCBA is positively related to employee psychological distress.

### Mediating Role of WFC

Through the lens of work–family boundary theory, time-based conflict may occur when the time spent on ICTs outside of work hours makes them no longer able to participate in their family roles and activities because individuals cannot allocate time to work and family time. Besides, the use of work-related ICT outside of working hours may trigger stress spillages from the work area to the home. Continuous connectivity makes it more difficult for people to dissociate and disengage from work at home, thus impeding the recovery of work stress outside of working hours (7), and stress-based conflict can happen.

Studies have shown that frequent WCBA induces ontogenesis of WFC (8). WFC leads to job dissatisfaction, turnover intention, and stress, and WFC plays a mediating role between the flexibility of work schedule and work stress (9). Liang and Chen (10) explored the influencing factors of fatigue among 3,603 employees of 35 Internet enterprises. They found that the use of Internet communication technologies and tools positively predicted the level of work stress. In contrast, occupational stress factors brought by work stress were negatively correlated with employees' mental health. Some scholars have further investigated the differences in WFC on employees of different genders and found that the traditional male and female division of labor still affects the amount of family role sharing. WFC is significant in predicting depression symptoms of female training physicians (11). Moreover, the psychological distress caused by WFC is higher for women than for male individuals (11). Therefore, we propose the following hypothesis:

*Hypothesis 2*: WFC has a mediating effect on WCBA and employee psychological distress.

### Moderating Role of Leader Workaholism

According to the previous studies, workaholism refers to the phenomenon of employees who become overindulging in work so that their health and family life are harmed (12). Based on this definition, this study defines a workaholic leader as individuals who are physically overworked and cognitively addicted to work. Workaholism tends to stimulate a tense and competitive work environment and develop negative interactions with employees. WCBA tends to cause resentment, anger, and other negative emotions in individuals, thus causing WFC (13). According to Andreassen et al. (14), leaders with high levels of workaholism tend to vent their negative emotions to their employees and therefore raise employees' aggression, which would increase negative emotional expression in the family and trigger WFC. In addition, workaholic leaders tend to give vague or unreasonable task requirements to employees, which consumes employees' time and energy, so employees of workaholic leaders are more likely to work overtime, which ultimately increases the possibility of WFC. A leader with high-level workaholism may strengthen the relationship between WCBA and WFC (15), which thereafter damages mental health. Therefore, we propose the following hypothesis:

*Hypothesis 3a*: Leader workaholism positively moderates the relationship between WCBA and WFC. When the leader's workaholism tendency is high, the positive prediction of WFC by WCBA is strengthened, whereas weakened.

However, the existing literature has not reached a consensus on whether workaholic leaders play a positive or negative moderating role between WCBA and WFC. Pan (16) found that when employees are encountering WFC, leader workaholism could provide necessary social support. Employees who experience high levels of social support at work (17) are more likely to continue working after working hours because they enjoy the interpersonal support and work assistance from colleagues. Therefore, the support brought by workaholic leaders may weaken the impact of WCBA on WFC. Thus, an auxiliary hypothesis is proposed:

*Hypothesis 3b*: Leader workaholism negatively moderates the relationship between WCBA and WFC. When the leader's workaholism tendency is high, the positive prediction of WFC by WCBA is weakened, whereas strengthened.

Combining hypotheses 2 and 3, a moderated mediating effect model is proposed:

*Hypothesis 4*: Leader workaholism moderates the indirect effect between WCBA and employee psychological distress *via* WFC. This indirect effect can be moderated by the leader's workaholic tendencies.

**Figure 1:**
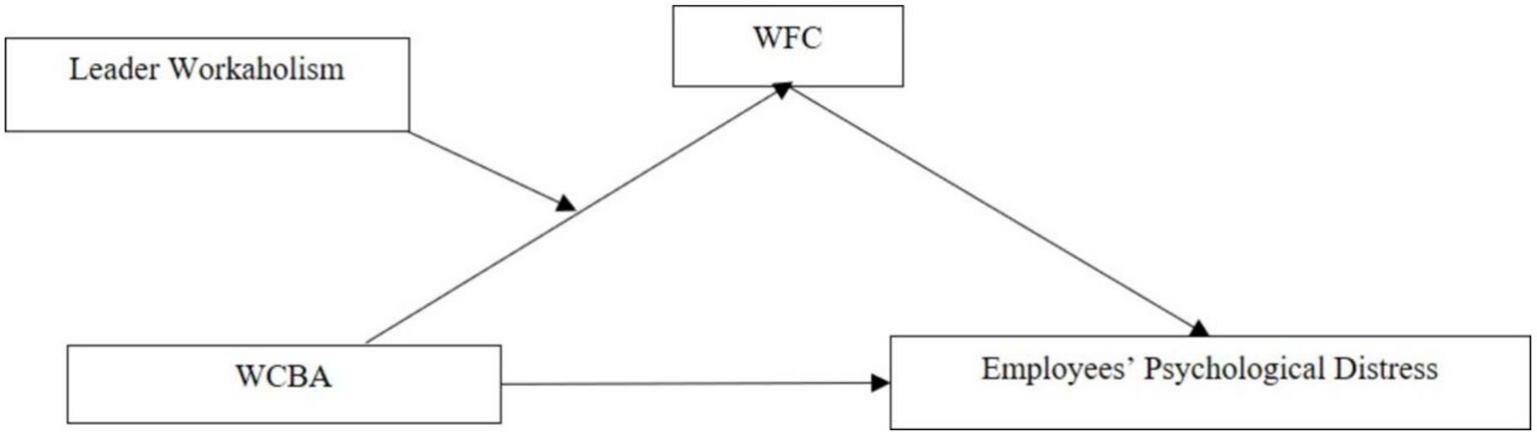
Research theoretical model.

The theoretical model of this study is shown in Figure 1.

## Methods

To test the hypothesis, we used the questionnaire method.

### Participants

Participants include both team leaders and their direct employees in four internet companies located in Beijing, Guangzhou, Shanghai, and Hangzhou.

### Procedure

The data were collected in two waves, from March to April in 2021. First, we contacted several HR managers in four internet companies to ask their support to release the paper questionnaires in their companies. The questionnaire contains demographic information and was attached with a letter explaining the purpose of the survey, voluntary participation, and guaranteed confidentiality. At time 1, the workaholism scale was sent to team leaders in four companies, and one direct team member of the leader rated his/her WCBA. At time 2 (three weeks later), these team members were recontacted to complete the scales of psychological distress and WFC. All scales were clearly explained with unified and standardized instructions given by researchers. A total of 1,200 questionnaires were sent in the first wave, which include 600 leader versions and 600 employees' versions. The second wave contained 600 questionnaires only for employees. All participants were asked to fill out the questionnaire and put it back into an envelope that was collected by our research team.

### Measurement

Each variable in the self-administered survey was measured using a multiitem scale, each of which was adopted from relevant prior research. As all our participants were Chinese, we followed the double-blind backtranslation procedure (18) to translate all items into Chinese. To avoid translation ambiguity, each item was translated by professional translators. The internal consistency of each scale was verified through Cronbach's alpha.

#### Work Connectivity Behavior After-Hours (WCBA)

Work connectivity behavior after-hours was assessed by a 13-item scale of Wu et al. (15) recommended by Richardson and Benbunan-Fich (19) who suggested collecting “lean” measures to capture activity (e.g., duration of use) and also “rich” measures that incorporate information about the nature of the activity (e.g., breadth of use, the context of use). Therefore, we collected self-reported measures of both duration (e.g., how much time they use the wireless devices) and frequency in context (e.g., how often they use the devices during particular non-work activities). Each item was measured on a 5-point Likert scale, ranging from 1, which indicates “never,” to 5, indicating “always.” First, we measured WCBA duration by asking respondents to report, on average, how much time they used each device (e.g., wireless email devices and laptops) to perform job-related duties during non-work hours. We collected responses for four time periods (e.g., before work, after work, during days off, and weekend and vacation). We provided response categories in ranges of minutes to create a Likert-type scale (e.g., 1–15 min, 16–30 min, et al.). The Cronbach's alpha was 0.830. Second, to create a measure for WCBA frequency, we followed Boswell and Olson-Buchanan (20), who asked respondents to report the frequency (on a Likert-type scale) with which they used an array of communication technologies to perform their job during non-work hours. Their study responses to the individual technologies were averaged to create an overall index of reported communication technology use after hours. To improve the reliability of Boswell and Olson-Buchanan's measure, we first asked about the use of a specific technological device (e.g., handheld wireless devices, laptops) rather than the communication medium (e.g., sending and receiving work emails, contacting colleagues or customers, logging in to the company website, etc.), and then, we asked how frequently each device is used during a specific non-work activity (e.g., shopping, travel/vacation, dinner, reading, fitness, etc.). We averaged the responses to the individual technologies to create an overall index of WCBA frequency. The Cronbach's alpha was 0.914, and the Cronbach's alpha of the total scale was 0.933.

#### Leader Workaholism

Workaholism was measured with a 10-item scale from the Chinese version of She et al. (21) and the original English scale from Schaufeli et al. (22). Workaholism was operationalized by two scales: WE, as assessed with the 5-item Compulsive Tendencies Scale of the WART (23); WC, as assessed with the 5-item drive scale of the WorkBat (24). Items were scored on a 5-point rating scale, which ranges from 1 (totally disagree) to 5 (totally agree). Sample items were “racing against the clock, continue to work after colleagues left, many irons in the fire, more time working than socializing, doing two or three things at a time, important to work hard.” Cronbach's alpha was 0.960.

#### Work-to-Family Conflict

Work-to-family conflict was assessed using the four-point scale of Carlson et al. (25). Six items were used to measure WFC. Chinese scholar Wu et al. (15) stated that the items included subscale based on time conflict and subscale based on stress conflict. Sample items were “due to various pressures at work, sometimes even when at home, I am not in the mood to do what I like.” Cronbach's alpha was 0.936.

#### Psychological Distress

Psychological distress (PD) was measured by General Health Questionnaire (GHQ-12), which was widely used to measure the psychological distress (26). The scale asks whether the respondent has experienced a particular symptom or behavior recently. Each item is rated on a four-point scale (less than usual, no more than usual, rather more than usual, or much more than usual), and it gives a total score of 12 or 36 based on the scoring method selected. The most common scoring methods are bimodal (0–0–1–1) and Likert scoring (0–1–2–3). Since the latter produces a more acceptable distribution of scores for parametric analysis (less skewed and less kurtosis), we used the Likert scoring style for this study. A higher score indicates a lower degree of mental health, in other words, a higher degree of psychological distress. Cronbach's alpha was 0.945.

#### Control Variables

According to previous studies, we also collected the following demographic variables as control variables: age, gender, education level, and tenure (4, 27).

### Analytical Approach

The software SPSS 24.0 was used to conduct fundamental analyses, which includes descriptive statistics and correlations for WCBA, WFC, psychological distress, and leader workaholism. All variables were computed, and descriptive statistics, namely mean (*M*) and standard deviation (*SD*), and correlations between variables were obtained. Mplus 7.4 software was used to establish structural equation models (SEMs). In this study, a bootstrapping analysis was conducted with WCBA as the independent variable, Psychological distress as the outcome variable, WFC as mediators, and leader workaholism as moderator, with 5,000 resamples to test a moderated mediation model and to calculate the 95% CIs. The number of subdimensions in each scale was unequal; thus, mean scores of the items were used for all observable variables in this study.

## Results

### Descriptive Results

After eliminating incomplete and invalid questionnaires, 211 groups of valid matching data were finally collected. That is to say, 422 questionnaires were recovered with an effective response rate of 77%. Participants come from Beijing, Guangzhou, Shanghai, and Hangzhou, where many concentrated Internet enterprises. The sample characteristics of subordinate employees are as follows: average age is 27.56 years (*SD* = 3.189), men account for 44.4%, women account for 55.6%. The average working year is 2.34 years (*SD* = 2.196). College degree or below accounts for 10.7%, a bachelor's degree accounts for 78.6%, and master's degree or above accounts for 10.7%. The sample characteristics of direct leaders are as follows: average age is 34.14 years (*SD* = 3.947), 51.7% men and 48.3% women. The average working year is 4.3 years (*SD* = 8.354). College degree or below accounts for 0%, a bachelor's degree accounts for 49.3%, master's degree or above accounts for 50.7%.

The average scores and the standard deviations of variables under study and their Pearson's correlations are presented in Table 1. Tenure was positively correlated with age (*r* = 0.65, *p* < 0.01) but not with WCBA and WFC. WCBA was positively correlated with WFC (*r* = 0.90, *p* < 0.01) and psychological distress of employees (*r* = 0.87, *p* < 0.01). WFC was positively correlated with psychological distress (*r* = 0.89, *p* < 0.01), which provided the basis for hypothesis testing. From the perspective of leadership, the age of leaders was positively correlated with their tenure, whereas leader workaholism was not correlated with the educational background, age, and marital status of leaders, but negatively correlated with the tenure of employees (*r* = −0.16, *p* < 0.05). In addition, leader workaholism was positively correlated with WCBA (*r* = 0.69, *p* < 0.01), WFC (*r* = 0.75, *p* < 0.01) and psychological distress (*r* = 0.69, *p* < 0.01). Multiple regression analysis is needed to confirm further the relationship among the variables between the leader and employees at the crossorganizational level. The mean, standard deviation, and correlation coefficient of each variable are shown in Table 1.

**Table 1:** Means, standard deviations, and correlations of the research variable.

**Variable**	**1**	**2**	**3**	**4**	**5**	**6**	**7**	**8**	**9**
**Individual level**									
1. Gender	1.00								
2. Age	−0.07	1.00							
3. Education	−0.03	−0.11	1.00						
4. Employee's tenure	−0.01	0.65^**^	−0.06	1.00					
5. Marriage	−0.02	0.15^*^	−0.03	0.07	1.00				
6. WCBA	0.00	−0.12	−0.13	−0.06	−0.02	1.00			
7. WFC	−0.02	−0.06	−0.12	−0.00	−0.01	0.90^**^	1.00		
8. Psychological distress	−0.02	−0.13	−0.10	−0.09	0.03	0.87^**^	0.89^**^	1.00	
9. leader workaholism	0.07	0.07	−0.16^*^	0.05	0.09	0.69^**^	0.75^**^	0.69^**^	1.00
*M*	1.55	27.56	2.010	2.340	1.650	2.821	2.543	2.631	3.54
*SD*	0.50	3.19	0.4	2.196	0.690	0.907	0.918	0.743	1.16
**Leadership level**	1	2	3	4	5	6			
1. Gender	1.00								
2. Age	−0.16^*^	1.00							
3. Leader's education	0.02	−0.10	1.00						
4. Leader's tenure	−0.10	0.46^**^	−0.08	1.00					
5. Marriage	0.04	0.21^**^	0.17^*^	0.07	1.00				
6. leader workaholism	0.02	0.04	−0.11	0.05	−0.02	1.00			
*M*	1.48	34.14	2.51	4.30	2.82	3.54			
*SD*	0.50	3.95	0.50	2.16	0.58	1.15			

*N = 211. WCBA, work connectivity behavior after-hours; WFC, work-to-family conflict*.

* *p < 0.05*.

** *p < 0.01*.

### Common Method Bias

To test the extent to which the models are affected by common method bias, Harman's one-factor test was adopted. The goodness-of-fit index of one-factor model is as follows (See Table 2): χ^2^ = 2,148.10, *df* = 799, TLI = 0.804, CFI = 0.814, SRMR = 0.070. The goodness-of-fit index of six-factor model is as follows: χ^2^ = 1,094.427, *df* = 764, RMSEA = 0.045, SRMR = 0.039, CFI = 0.955, TLI = 0.952. The goodness-of-fit index of the six-factor model was far better than that of the one-factor model, which suggested that common method variance had less influence on this research. Besides, the six-factor model was far better than that of the other models as well, which suggested that our model has good discriminant validity.

**Table 2:** Fit indices of each model.

**Model**	**Factor**	**χ^2^**	***df***	**χ^2^/** ***df***	**RMSEA**	**SRMR**	**CFI**	**TLI**
Six factors model	A1,A2,B1,B2,C,D	1,094.427	764	1.432	0.045	0.039	0.955	0.952
Five factors model	A1+A2,B1,B2,C,D	1,140.215	772	1.477	0.048	0.039	0.950	0.947
Four factors model	A1+A2,B1+B2,C,D	1,145.751	776	1.476	0.048	0.040	0.950	0.947
Three factors model 1	A1+A2+C, B1+B2,D	1,820.138	776	2.346	0.080	0.083	0.858	0.851
Three factors model 2	A1+A2,B1+B2+D,C	1,207.577	779	1.550	0.051	0.041	0.942	0.939
One factor model	A1+A2+B1+B2+C+D	2,148.505	779	2.758	0.091	0.070	0.814	0.804

*N = 211; A = Work connectivity behavior after-hours (A1 and A2 represent two dimensions: duration and frequency); B = work-to-family conflict (B1 and B2 represent two dimensions: conflict over time and conflict overstress); C = leader workaholism; D = psychological distress*.

### Stepwise Regression of the Study Variables

Stepwise regression analysis was first conducted to test the hypothesizes, and Table 3 summarizes the result of the regression. We first entered control variables that include several demographic information. There is no significant impacts of age and gender on overtime work. The second step introduced WCBA and found that WCBA had a significant regression prediction for WFC (β = 0.901, *p* < 0.01). In the third step, we introduced leader workaholism and found that the leader workaholism could significantly predict WFC (β = 0.238, *p* < 0.01), and the regression coefficient between WCBA and WFC changed under the leader workaholism (β = 0.733, *p* < 0.01). The fourth step examined the interaction effect between WCBA and leader workaholism. The results showed that leader workaholism had a significant negative moderating effect on the relationship between WCBA and WFC, and the interaction term was significantly negative (β = −0.618, *p* < 0.01). Models 2, 3, and 4 combined to prove that leader workaholism played a weakening moderating role between WCBA and WFC, that is, with the increase of leader workaholism, the predicted value of WCBA to WFC decreased, which prove that hypothesis 3a is false and hypothesis 3b is supported. Model 6 showed that WCBA could significantly positively predict the psychological distress of employees (β = 0.871, *p* < 0.01), and Model 7 showed that WFC also significantly positively predicted the psychological distress of employees (β = 0.580, *p* < 0.01), which confirms that WFC could play a mediating role in the relationship between leader workaholism and psychological distress of employees. Both Hypotheses 1 and 2 were supported.

**Table 3:** Summary of stepwise regression analysis.

	**WFC**				**Psychological distress**		
	**Model 1**	**Model 2**	**Model 3**	**Model 4**	**Model 5**	**Model 6**	**Model 7**
Control variables							
Employee's gender	0.089	0.027	0.032	0.04	0.037	−0.023	−0.039
Employee's age	−0.100	0.041	0.004	0.003	−0.141	−0.005	−0.029
Employee's education	−0.128	0.001	0.012	0.016	−0.113	0.011	0.011
Employee's tenure	0.056	0.023	0.028	0.006	−0.009	−0.041	−0.054
Employee's office term	0.003	0.003	−0.015	−0.022	0.043	0.044	0.042
The independent variables							
WCBA		0.901^**^	0.733^**^	1.092^**^		0.871^**^	0.349^**^
The moderating variables							
Leader workaholism			0.238^**^	0.543^**^			
The interaction effect							
WCBA*leader workaholism				−0.618^**^			
The intervening variable							
WFC							0.58^**^
*R^2^*	0.030	0.808	0.835	0.842	0.034	0.761	0.826
Δ*R^2^*	0.030	0.508^**^	0.805^**^	0.812^**^	0.034	0.727^**^	0.792^**^
*F*	1.267	142.889^**^	147.001^**^	134.977^**^	1.425	108.556^**^	137.767^**^

*N = 211.The regression coefficient β is the standardized coefficient*.

** *p < 0.01*.

### Moderating Effect Testing

**Figure 2:**
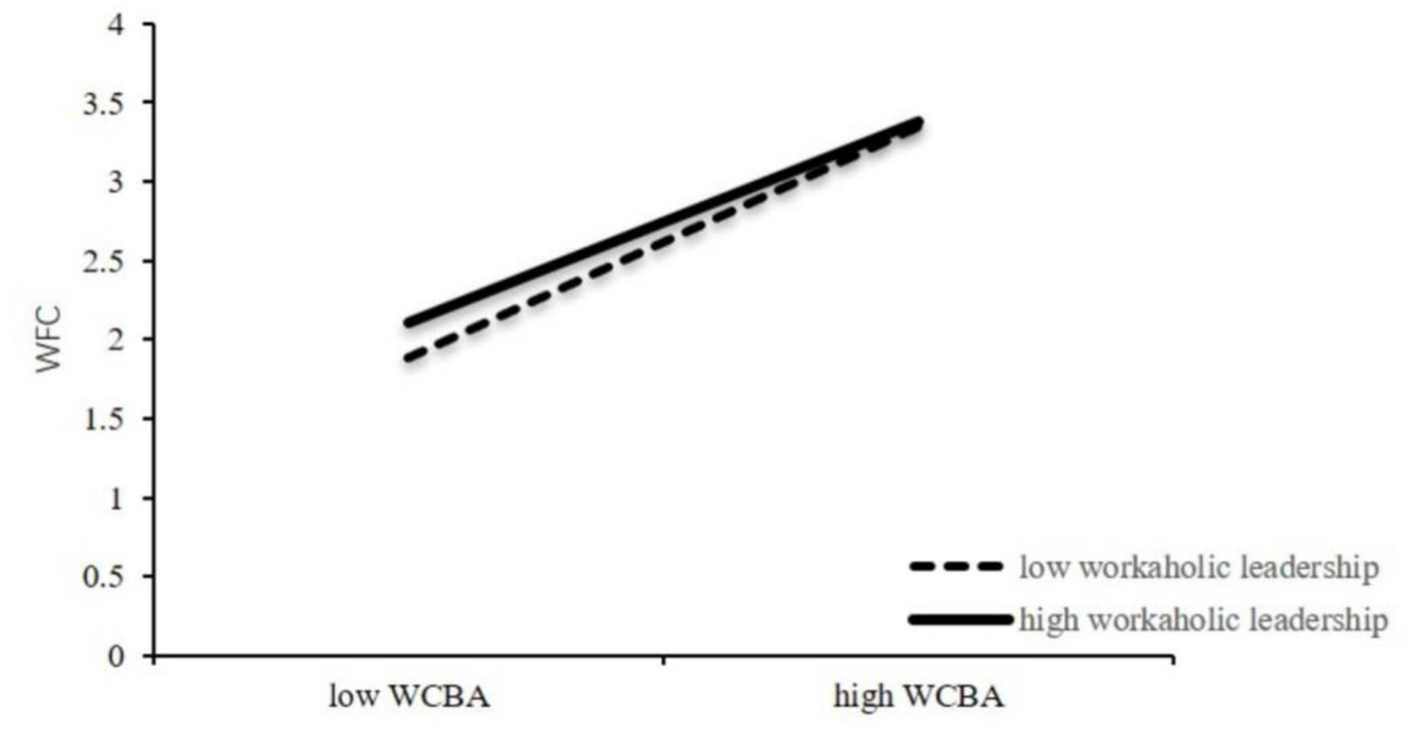
The moderating effects of leader workaholism on WCBA and WFC.

For the study's accuracy, the moderating effect test was then carried out. In addition, the product of the WCBA index and leader workaholism index was used as the interaction index, and the moderating effect of the product was tested. The results showed that the interaction between WCBA and leader workaholism could significantly predict WFC (β = −0.12, *p* < 0.01), as shown in Table 4. F test showed significant regression equation (*R*^2^ = 0.84, *F* = 362.52, *p* < 0.001). A simple slope test was conducted, a moderating effect graph was made, and the results are shown in Figure 2. The slope of the interaction term was negative, which proves that leader workaholism had a weakening moderating effect in the relationship between WCBA and WFC. As the tendency of leader workaholism becomes higher, the positive correlation between WCBA and WFC still exists, but it is weaker than before. Hypothesis 3b was supported again.

**Table 4:** The moderating role of leader workaholism.

**Variable**	**WFC**		**WFC**	
	**β**	***t***	**β**	***t***
WCBA	0.73^***^	18.62^***^	0.73^***^	18.79^***^
Leader workaholism	0.24^***^	6.04^***^	0.16^***^	3.54^***^
Product interaction term			−0.12^**^	−3.02^**^
*R^2^*	0.83^***^		0.84^**^
*F*	518.92^***^		362.52^***^

** *p < 0.01*,

*** *p < 0.001*.

### Mediating Effect Testing

The mediating effect of WFC was tested by SEM using Mplus 7.4 with WCBA as an independent variable, employees' psychological distress as a dependent variable, and WFC as a mediator. Basic model A represents the direct effect of WCBA on employees' psychological distress, and Model B represents the effect of WCBA on employees' psychological distress with WFC as the mediator.

The model fitting index is shown in Table 5. The mediating model (model B) fit well (TLI = 0.96, CFI = 0.96, RMSEA = 0.05, SRMR = 0.04). Table 6 summarized the indirect effect test result. The direct and indirect effects of WCBA on employees' psychological distress were 0.306 (*p* < 0.001) and 0.628 (*p* < 0.001), which account for 32.76% and 67.24%, respectively, in the total effect. The results of the Bootstrap analysis show that the fitting data of the main models in this study are good. The Bootstrap analysis results showed that the 95% confidence interval of the path in Model B was [0.899, 0.976], excluding 0, which indicates a significant partial mediating effect of WFC on the WCBA-psychological distress relationship.

**Table 5:** The model fitting index of the mediating effect.

**Model**	**χ^2^**	***df***	**χ^2^/** ***df***	**CFI**	**TLI**	**RMSEA**	**SRMR**
A	394.70	272	1.45	0.97	0.96	0.05	0.04
B	630.22	430	1.46	0.96	0.96	0.05	0.04

**Table 6:** Indirect effect of WFC.

**Item**	**Point estimate**	**Product of coefficients**			**BC 95% CI**	
		**S.E**.	**Est./S.E**.	***P*** **-Value**	**Lower**	**Upper**
Total	0.934	0.021	44.162	*p* < 0.001	0.899	0.976
Total direct	0.306	0.021	14.737	*p* < 0.001	0.265	0.306
Total indirect	0.628	0.021	29.555	*p* < 0.001	0.593	0.663

*BC 95% CI, bias-corrected 95% bootstrap confidence interval*.

### Moderated Mediation Effects Testing

**Figure 3:**
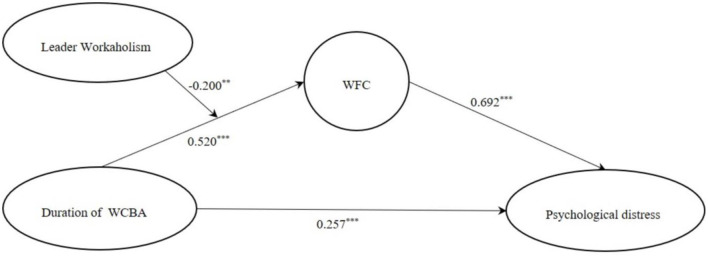
Model path diagram based on the duration of WCBA.

**Figure 4:**
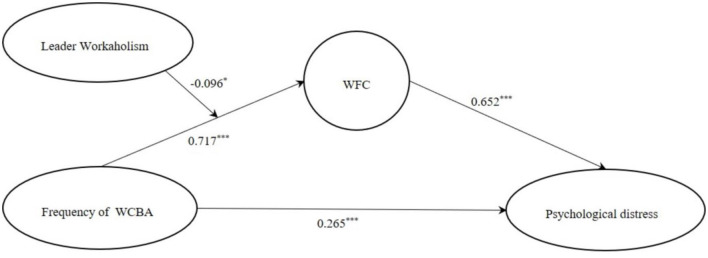
Model path diagram based on the frequency of WCBA.

Mplus7.4 was used again to justify the moderated mediation model with Bootstrap. WCBA path diagrams based on duration and frequency are shown in Figures 3, 4.

With arrows that indicate the direction in which the antecedent variable acted on the outcome variable, from the figure, we could see the direct effect and indirect effect among the variables, and the path coefficient represented the strength of the correlation between the variables.

However, only the path diagram was far from enough, and SEM fitting index test was a vital link. Based on generalized least squares estimation (GLS), the following indexes were used as the model fitting indexes: TLI > 0.9, RMSEA < 0.08, CFI > 0.9, SRMR < 0.08, which proves that the moderated mediating effect model in this study has a good fitting degree. Thus, Hypothesis 4 was supported. It was found that WCBA positively predicted WFC. With the increase of leader workaholism, the positive predictive effect of WCBA on WFC was weakened (but the two were still positively correlated), which might be related to the double characteristics of leader workaholism with high job requirements and high job involvement:

First, the high work requirements of workaholic leaders make workaholic leaders highly positively correlated with WCBA and WFC.

Second, the high work commitment of workaholic leaders provides resource support for employees. With the continuation of WCBA, workaholic leaders will provide more work support, such as providing problem-solving ideas and delegating colleagues to share the work, etc., which alleviates subordinate WFC.

Finally, whether from the perspective of high job requirements or high job involvement, the workaholic leader will provide work support and help to the subordinates to ensure the timely completion of the project. The higher the degree of workaholism, the more resource support employees get, which weakens the positive predictive value of WCBA to WFC. The research results are not only consistent with the resource conservation theory but also consistent with the research results of Pan (16).

## Discussion

This study reveals that WCBA can positively predict employees' psychological distress, and WFC plays a mediating role in the relationship between WCBA and employees' psychological distress. Leader workaholism negatively moderates the relationship between WCBA and WFC. When the leader's workaholism tendency is high, the positive prediction of WFC on WCBA is weakened, and vice versa. Furthermore, leader workaholism moderates the indirect effect of WFC, that is, the indirect effect is stronger when the leader's workaholic tendencies are lower.

### Theoretical Implications

First, we again verified that WCBA positively predicts employees' psychological distress, which is consistent with previous studies (1–3). In addition, past questions about WCBA and psychological distress mechanisms have mainly been approached from the perspective of individual cognitions (28, 29). However, environmental factors, especially family environmental factors, are also essential for employees' mental health (9, 11). Therefore, we filled this gap by verifying the mediating effect of WFC in WCBA and individual psychological distress from the perspective of work–family boundary theory. Our findings expand the scope of work–family boundary theory in explaining organizational context factors and individual psychological distress. In the future, other variables of the work–family boundary can be further explored in the relationship of WCBA and employees' psychological distress, such as work–family balance. Finally, we explored the boundary conditions of this mechanism. We obtained an interesting finding that the degree of workaholism of the leader is an important moderator. When the degree of workaholism of the leader is high, it can weaken the predictive effect of WCBA on WFC, thus decreasing the negative predictive effect on employees' mental health. A leader's behavior and attitude to work can cause spread step-by-step and transfer to the subordinates. Our study further enriches the empirical research literature on the transmission effect of leadership.

### Practical Implications

Our study showed the negative predictive effect of WCBA on employees' mental health. Therefore, at the individual level, individuals should participate in life activities appropriately after work, increase the transition activities between work and non-work fields to promote psychological detachment, and maintain mental health. At the organizational level, managers should make reasonable use of human resource management theory, scientifically design the working process to avoid employees' WCBA, which is conducive to alleviating employees' job burnout (30).

We found that WFC is the mediating mechanism of predicting the effect of WCBA on employees' mental health. Thus, when some work tasks have to be done during family time, the company should give enough support to employees, especially the support to the family, to reduce the impact of work on the family. For example, the enterprise can hold family-orientation activities to give employees much more time to spend with family.

Leader workaholism will bring serious negative predictive effect on employees' physical and mental health, which is not conducive to the organization's long-term development. Interestingly, however, our findings suggested that leader workaholism negatively moderates the relationship between WCBA and WFC. It is the resources to complete the work from workaholic leader that weaken the negative predictive effect of WCBA; thus, leaders need to provide problem-solving resources during employees' non-working hours.

### Limitations and Future Research

From the theoretical perspective, it is suggested to consider in the future: First, experimental design, such as laboratory experiments or intervention studies, should be introduced to clarify further the influence of WCBA on individuals and its mechanism of action. Second, we suggest that subsequent researchers combine field studies, quasiexperimental studies, case studies, and in-depth interviews to explore the deeper relationship between WCBA and individual psychological distress or organizational performance. Third, in terms of data collection, on the one hand, longitudinal timing design should be combined; on the other hand, self-evaluation of the subjects should be combined with other objective evaluations, such as using the APP to record the time and frequency of WCBA. Fourth, we suggest considering the forced selection scale measurement to distinguish the workaholic and non-leader workaholism in the statistical analysis method. Last, considering that our results are based on the Chinses sample, cross country studies are needed to test the stability of our results.

From the empirical perspective, besides the moderating effect of leader workaholism, job characteristics and individual subjective will may also present the pathway function of non-working time connecting to WFC. Considering only leader workaholism as a moderating variable is a little monotonous, which can be improved in the future. What is more, to enrich the transmission path between leaders and subordinates, more variables such as job characteristics (organizational assessment and workload) and leadership style (such as authoritative leadership) can be explored in the future.

## Data Availability Statement

The raw data supporting the conclusions of this article will be made available by the authors, without undue reservations.

## Ethics Statement

The studies involving human participants were reviewed and approved by this study was carried out in accordance with the recommendations of the Ethics Committee of the Department of Psychology, Renmin University of China, with written informed consent from all subjects. All subjects gave written informed consent in accordance with the Declaration of Helsinki. The protocol was approved by the Ethics Committee of the Department of Psychology, Renmin University of China. The patients/participants provided their written informed consent to participate in this study.

## Author Contributions

MD designed and drafted the work. TZ made the main revision for the study and replied to reviewers. YL revised the original manuscript and the revisions. ZR collected the data. All authors contributed to the article and approved the submitted version.

## Funding

The study was supported by Major Innovation and Planning Interdisciplinary Platform for the Double-First Class Initiative, Renmin University of China.

## Conflict of Interest

The authors declare that the research was conducted in the absence of any commercial or financial relationships that could be construed as a potential conflict of interest.

## Publisher's Note

All claims expressed in this article are solely those of the authors and do not necessarily represent those of their affiliated organizations, or those of the publisher, the editors and the reviewers. Any product that may be evaluated in this article, or claim that may be made by its manufacturer, is not guaranteed or endorsed by the publisher.
