# Supplementary material for: The Effect of Work Connectivity Behavior After-Hours on Employee Psychological Distress: The Role of Leader Workaholism and Work-to-Family Conflict
Source: Front Public Health. 2022 Feb 23;10:722679. doi: 10.3389/fpubh.2022.722679 (PMC8905642; doi:10.3389/fpubh.2022.722679)
Supplement: Supplementary file 1 [file Data_Sheet_1.zip › 722679-supplementary materials/722679-questionnaire.pdf]

The appendix

1 Leader Workaholism

Racing against the clock.
Continue to work after colleagues left
Many irons in the fire
More time working that socializing
Doing two or three things at a time
Important to work hard
Something inside me that drives me
Feel obliged to work hard
Feel guilty when take time off work
Hard to relax when not working

2 Information filling of interviewees

Your gender:
Your age is ___
Your education background:
Working length in current position: _____ years
Your marriage and child status:

3 Mental Health Survey Questionnaire for the Internet Industry

3.1 the usage frequency of the connected tool during non-working hours

3.1.1 During the following non-working hours, what is the average time you use mobile devices (such as mobile phone/laptop/tablet, etc.) to deal with work affairs or communicate with colleagues? (every day)

Duration of handling work affairs or communicating with colleagues during lunch break:
Duration of handling work affairs or communicating with colleagues after work:
Duration of handling work affairs or communicating with colleagues on weekends:
Duration of handling work affairs or communicating with colleagues on holidays:

3.1.2 How often do you use mobile communication devices to deal with work affairs (such as sending and receiving work emails, contacting colleagues or customers, logging in to the website of the company/company, etc.) during the following activities?

Commuting
Exercise
Shopping
Travel/Vacation
Eat (at home or out)
Reading/surfing the Internet
A film/play/concert

A party or social event

Courses

3.2 Work-to-family conflict

Please choose the option that is more in line with your normal state according to the actual situation. The content you fill in will be kept confidential and not visible to your supervisor.

I have to miss some family events because of my work.

Because I have to devote a lot of time to my work, I can’t share some of my family responsibilities.

My job takes away time that I should be spending with my family and friends.

When I get home from work, I’m often too emotionally consumed by work to do anything for my family.

Because of all the pressure at work, sometimes I’m not in the mood to do what I like even when I’m relaxing at home.

After coming home from work, I often feel very tired and don’t want to participate in family activities or do housework and so on.

3.3 Mental health status

Can you concentrate on whatever you are doing?

Insomnia due to anxiety

Do you feel that things are working?

Do you feel able to make up your mind about things?

I feel nervous all the time

Feel unable to overcome difficulties

Can you enjoy your daily activities?

Can you avoid conflict?

Feeling unhappy and depressed

Have you lost confidence in yourself?

Think of yourself as a worthless person

On the whole, do you feel moderately happy?
